# Efficacy of immune checkpoint inhibitor treatment for head and neck mucosal melanoma recurrence in patients treated with carbon‐ion radiotherapy

**DOI:** 10.1002/cnr2.1825

**Published:** 2023-04-28

**Authors:** Atsushi Musha, Nobuteru Kubo, Hidemasa Kawamura, Naoko Okano, Hiro Sato, Kohei Okada, Kento Tomizawa, Norichika Ota, Akiko Adachi, Masato Shino, Osamu Nikkuni, Shota Ida, Katsuyuki Shirai, Jun‐ichi Saitoh, Satoshi Yokoo, Kazuaki Chikamatsu, Tatsuya Ohno

**Affiliations:** ^1^ Gunma University Heavy Ion Medical Center Maebashi Japan; ^2^ Department of Oral and Maxillofacial Surgery and Plastic Surgery Gunma University Graduate School of Medicine, Maebashi Maebashi Japan; ^3^ Department of Otolaryngology‐Head and Neck Surgery Gunma University Graduate School of Medicine Maebashi Japan; ^4^ Department of Radiology Jichi Medical University Hospital Tochigi Japan; ^5^ Division of Radiation Oncology, Department of Radiology Faculty of Medicine, University of Toyama Toyama Japan

**Keywords:** chemotherapy, melanoma, radiotherapy, survival

## Abstract

**Background:**

Carbon‐ion radiotherapy (C‐ion RT) is effective for head and neck mucosal melanoma (HN‐MM), including radioresistant mucosal melanoma. Melanoma also responds effectively to immune checkpoint inhibitors (ICIs). Data on the efficacy and safety of ICIs for HN‐MM are insufficient.

**Aims:**

To analyze the efficacy and safety of ICI salvage therapy in patients with HN‐MM recurrence after C‐ion RT.

**Methods and Results:**

This retrospective study analyzed the medical records of 52 patients with HN‐MM treated with C‐ion RT between 2012 and 2020. A dose of 57.6 or 64.0 Gy (relative biological effectiveness) was provided in 16 fractions. The primary endpoint was 3‐year overall survival (OS) rate. The median follow‐up time was 26.8 months for all patients. A total of 29 patients had local recurrence or distant metastasis, and 16 patients who received ICI therapy. The 3‐year OS rate in the ICI group (*n* = 16) and best supportive care group (*n* = 13) were 53.8% and 0.0%, respectively (*p* = 0.837); the difference was not statistically significant. There were no deaths after 1 year among patients who underwent ICI therapy. No adverse events associated with C‐ion RT were related to or exacerbated by ICI.

**Conclusion:**

ICI salvage therapy is effective and safe for patients with HN‐MM recurrence after C‐ion RT.

## 1 INTRODUCTION

The United States National Cancer Database reported a cutaneous melanoma incidence of over 90%, with mucosal melanomas accounting for 1.3% of onsets at the head and neck.^1^ Head and neck mucosal melanomas (HN‐MM) have a very poor prognosis because of distant metastases, with a 5‐year overall survival (OS) rate of approximately 20–30% after surgery, radiotherapy (RT), and chemotherapy.^1^, ^2^, ^3^, ^4^ Surgery and postoperative RT are the mainstays in treatment in most cases of HN‐MM.^2^, ^3^ However, surgery is not feasible in all cases, owing to wide resection margins and difficulty in reconstruction, which could result in cosmetic and functional challenges that impact the patient's quality of life.^5^

Mucosal melanoma has been considered radioresistant; however, there is evidence supporting the benefits of RT. A retrospective multi‐center study by the Japan Carbon‐Ion Radiation Oncology Study Group (J‐CROS) reported a 5‐year OS rate of 44.6% and a local control (LC) rate of 72.3% in patients with HN‐MM after carbon‐ion (C‐ion) RT, indicating that it is one of the most effective treatment modalities.^6^ In a prospective study, HN‐MM treated with C‐ion RT and dacarbazine, nimustine, and vincristine (DAV) had a 3‐year OS rate of 49.2% and an LC rate of 92.3%. C‐ion RT had a reproducible effect on HN‐MM; however, the most distant metastases were recurrent, with an approximately 1‐year median duration of recurrence. There were no significant cases with progression‐free survival (PFS) and OS after the completion of 3 cycles of therapy with or without DAV.^7^

The previous study showed that cancers, such as melanoma and lung, had the highest prevalence of somatic mutation and responded best to immune checkpoint inhibitors (ICIs).^8^ Advanced melanoma was shown to have a 5‐year OS of 52% in a treatment group of nivolumab plus ipilimumab, 44% in a treatment group of nivolumab, and 26% in a treatment group of ipilimumab.^9^ Furthermore, ICIs and RT are thought to play a significant role in the activation of immune responses.^10^ At present, recurrence of HN‐MM after C‐ion RT is treated with ICIs. However, both of their efficacy and safety are insufficient. Therefore, we arranged a retrospective study to evaluate the safety and efficacy in recurrent HN‐MM after C‐ion RT.

## 2 METHODS

### 2.1 Patients

**FIGURE 1:**
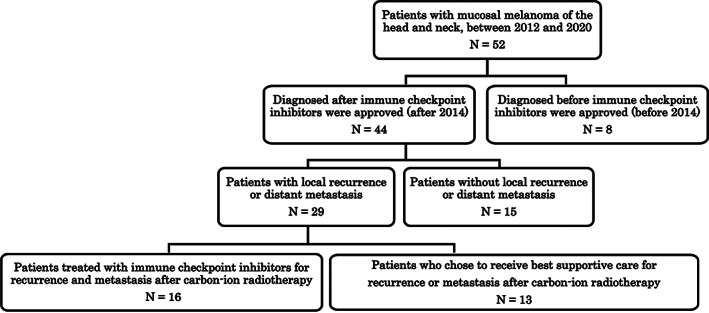
Flow chart of patients with mucosal malignant melanoma treated with carbon‐ion radiotherapy.

This retrospective clinical study enrolled 52 patients with HN‐MM (29 males and 23 females) treated with C‐ion RT between August 2012 and November 2020 at Gunma University Heavy Ion Medical Center. The median age was 71.5 (range of 32–91). This study was approved by the Gunma University Review Board (trial approval number: HS2021‐238) and was conducted in accordance with the Declaration of Helsinki. All patients provided written consent prior to C‐ion RT. The inclusion criteria were (a) histologically confirmed HN‐MM; (b) clinical stage of T1‐4N0M0, according to the Union for International Cancer Control staging criteria, 8th edition; (c) measurable tumor; (d) 20 years of age or older; (e) no abnormal cardiac, pulmonary, hepatic, or bone marrow function; and (f) performance status from 0 to 2. The exclusion criteria were (a) history of RT on the head and neck region; (b) history of chemotherapy in the 4 weeks before C‐ion RT administration; (c) severe infection; (d) severe clinical complications; and (e) active multiple primary cancer. The included patients underwent laryngoscopy by a physician and medical imaging, such as computed tomography (CT), magnetic resonance imaging (MRI), and F‐18 fluorodeoxyglucose positron emission tomography/CT performed within 1 month before C‐ion RT. We categorized patients into groups based on the time of diagnosis (before vs. after 2014, when ICIs were approved) and the presence of recurrence or distant metastasis (Figure 1).

### 2.2 Carbon‐ion RT procedure

The C‐ion RT technique and treatment planning methods used in this study were previously reported.^7^, ^11^, ^12^ Patients in personalized cradles (Moldcare; Alcare, Tokyo, Japan) were immobilized using thermoplastic shells (Shellfitter; Kuraray, Osaka, Japan) and positioned. A personalized mouthpiece immobilized the mandible.^13^ CT simulation (thickness of 2 mm) was used for C‐ion RT planning and MRI for baseline reference. The XiO‐N system (Elekta, Stockholm, Sweden) was performed for treatment planning. Contouring for the gross tumor volume (GTV) was used as a reference contrast‐enhanced MRI image. The clinical target volume (CTV) criterion is a 5 mm margin around the GTV for all directions and used as reference of anatomical structure. The planning target volume (PTV) had 2 mm margins around the CTV. The target volumes of the clinical and C‐ion RT planning margins were modified as necessary when the targets were close to the organs at risk (OAR), such as optic nerve, eye, brain, and brain stem. CTV and PTV were modified to protect OAR. The modification rate was 80.8% (42 of 52 cases). The nasal cavity and paranasal sinuses tended to be invaded to the proximity of the above OARs, which accounted for the majority of cases of correction. Calculations of the physical dose were based on the algorithm of the pencil‐beam, and those of the clinical dose distribution were performed using the physical dose and the relative biological effectiveness (RBE), used as reference of human submandibular gland cells.^14^ The C‐ion RT dose was expressed as “Gy (RBE),” 16 fraction size, and the overall treatment time was approximately 4 weeks (four fractions per week). Generally, C‐ion RT was treated by 64.0 Gy (RBE). When the mucosa and skin were widely irradiated, 57.6 Gy (RBE) was selected. Eight patients received 57.6 Gy (RBE) and 44 patients received 64.0 Gy (RBE).

Thirty‐six patients with HN‐MM received three courses of chemotherapy (day 1: 120 mg/m^2^ dacarbazine, 70 mg/m^2^ nimustine, and 0.7 mg/m^2^ vincristine; days 2–5: 120 mg/m^2^ dacarbazine) at 4‐week intervals, the first course at the start of C‐ion RT, second course after the end of C‐ion RT, and last course thereafter. The adverse events were categorized according to the Common Terminology Criteria for Adverse Events v4.0.

### 2.3 Follow‐up

For the first 6 months, follow‐ups for patients were every month, and every 3 months thereafter. Medical imaging, such as CT and MRI, were performed alternately every 2–3 months, and F‐18 fluorodeoxyglucose positron emission tomography/CT was performed every year. All patients, including those who developed lymph node or distant metastases, were assessed for LC during follow‐up until death.

### 2.4 ICI therapy

Treatment after C‐ion RT was not prescribed. However, individuals who received ICIs at recurrence were identified through retrospective medical record review. The following ICIs were considered: nivolumab and pembrolizumab (anti‐apoptotic, anti‐programmed death 1 [PD‐1]), ipilimumab (anti‐cytotoxic T‐lymphocyte‐associated protein 4 [CTLA4]), and their combinations. These drugs were administered until disease progression or unacceptable toxicity. The exposure cohort was patients who received ICI salvage therapy for HN‐MM recurrence after C‐ion RT and the control cohort was patients who did not. In Japan, ICI agents were approved for the treatment for melanoma patients in 2014.

### 2.5 Endpoints

Three‐year OS was the primary endpoint. LC rate, PFS, and adverse event occurrence were secondary endpoints.

### 2.6 Statistical analysis

LC, OS, and PFS rates with C‐ion RT were estimated by the Kaplan–Meier method and compared by log‐rank test. Between‐group differences were evaluated by t‐test. Differences were considered statistically significant at *p* < .05. Statistical analyses were performed with IBM SPSS version 26.0 (IBM Corp., Armonk, NY, USA).

## 3 RESULTS

**FIGURE 2:**
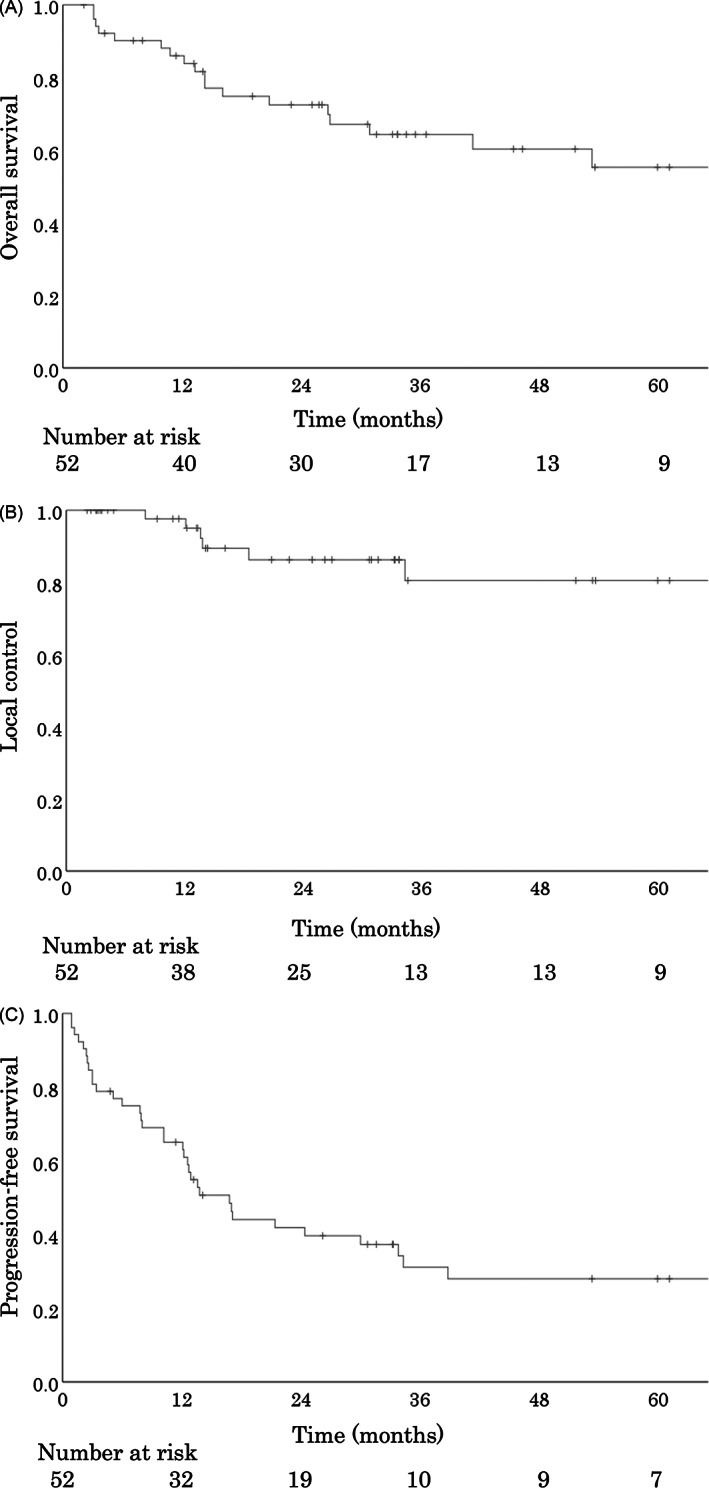
OS, LC, and PFS curves of patients with mucosal malignant melanoma treated with carbon‐ion radiotherapy. (A) The 3‐year OS rate for all patients (*n* = 52) was 64.3%. (B) The 3‐year LC rate for all patients (*n* = 52) was 86.3%. (C) The 3‐year PFS rate for all patients (*n* = 52) was 31.1%. LC, local control; OS, overall survival; PFS, progression‐free survival.

Table 1 shows the patient characteristics. The nasal cavity (*n* = 39), paranasal sinus (*n* = 5), oral cavity (*n* = 5), and others (*n* = 3) were primary tumor sites. The median follow‐up time for all patients was 26.8 (range, 2.1–111.7) months. Figure 1 shows the patient flow chart for this study. The 3‐year OS, LC, and PFS rate for all patients (*n* = 52) were 64.3% (95% confidence interval [CI], 53.9%–83.0%) (Figure 2A), 86.3% (95% CI, 73.2%–103.8%) (Figure 2B), and 31.1% (95% CI, 25.2%–49.8%) (Figure 2C), respectively. No cases of local recurrence due to modification of CTV or PTV to protect OAR were identified.

**TABLE 1:** Patient and tumor characteristics.

Characteristic	
Total (*n*)	52
Age (y), mean (range)	71.5 (32–91)
Sex, *n* (%)	
Male	29 (56)
Female	23 (44)
Histological type, *n* (%)	
Malignant melanoma	52 (100)
Region, *n* (%)	
Nasal cavity	39 (75)
Paranasal sinus	5 (10)
Oral cavity	5 (10)
Others	3 (5)
T stage, *n* (%)	
T3	4 (8)
T4a	40 (77)
T4b	5 (10)
X	3 (5)
N stage, *n* (%)	
N0	50 (96)
N1	2 (4)
M stage, *n* (%)	
M0	52 (100)
GTV (ml), mean (range)	16.4 (1.2–114.6)
Performance Status (%)	
0	14 (27)
1	38 (73)
Total dose, *n* (%)	
57.6Gy(RBE)	8 (15)
64.0Gy(RBE)	44 (85)
DAV therapy, *n* (%)	
DAV	36 (69)
None	16 (31)
ICI, *n* (%)	
ICI	16 (31)
None	36 (69)

Abbreviations: GTV, gross tumor volume; DAV, dacarbazine, nimustine, and vincristine; ICI, immune checkpoint inhibitor; RBE, relative biological effectiveness.

Adverse events after C‐ion RT are summarized in Table 2. Acute grade 2 mucositis and grade 1 dermatitis were common adverse events, which improved by conservative therapy. Leukopenia, anemia, and thrombocytopenia were improved after DAV therapy. One patient required analgesia due to late effect grade 4 mucositis. Another patient had grade 4 oral fistula due to tumor invasion of the maxilla and required a dental prosthesis. Two patients required hyperbaric oxygen therapy and analgesia due to grade 3 and 4 osteoradionecrosis.

**TABLE 2:** Acute and late adverse events in patients treated with carbon‐ion radiotherapy (n = 52).

	Any grade	Grade 1	Grade 2	Grade 3	Grade 4
Acute					
Mucositis	49	9	36	4	‐
Dermatitis	48	30	18	–	–
Xerostomia	12	8	4	–	–
Dysgeusia	7	4	3	–	–
Conjunctivitis	18	10	8	–	–
Leukopenia	28	14	7	7	–
Anemia	27	24	3	–	–
Thrombocytopenia	36	22	11	3	–
Late					
Mucositis	15	6	8	–	1
Dermatitis	20	19	1	–	–
Xerostomia	12	12	–	–	–
Dysgeusia	5	5	–	–	–
Conjunctivitis	4	3	1	–	–
Trismus	3	3	–	–	–
Oral fistula	6	1	4	–	1
Osteoradionecrosis	8	–	6	1	1
Brain necrosis	–	–	–	–	–
Brainstem necrosis	–	–	–	–	–
Nasal congestion	24	14	10	–	–
Chronic sinusitis	6	4	2	–	–
Olfactory nerve disorder	5	3	2	–	–
Middle ear infection	8	2	6	–	–
External otitis	–	–	–	–	–
Intraocular hemorrhage	1	1	–	–	–
Cataract	–	–	–	–	–
Optic nerve disorder	1	1	–	–	–
Nasolacrimal duct obstruction	14	14	–	–	–

**FIGURE 3:**
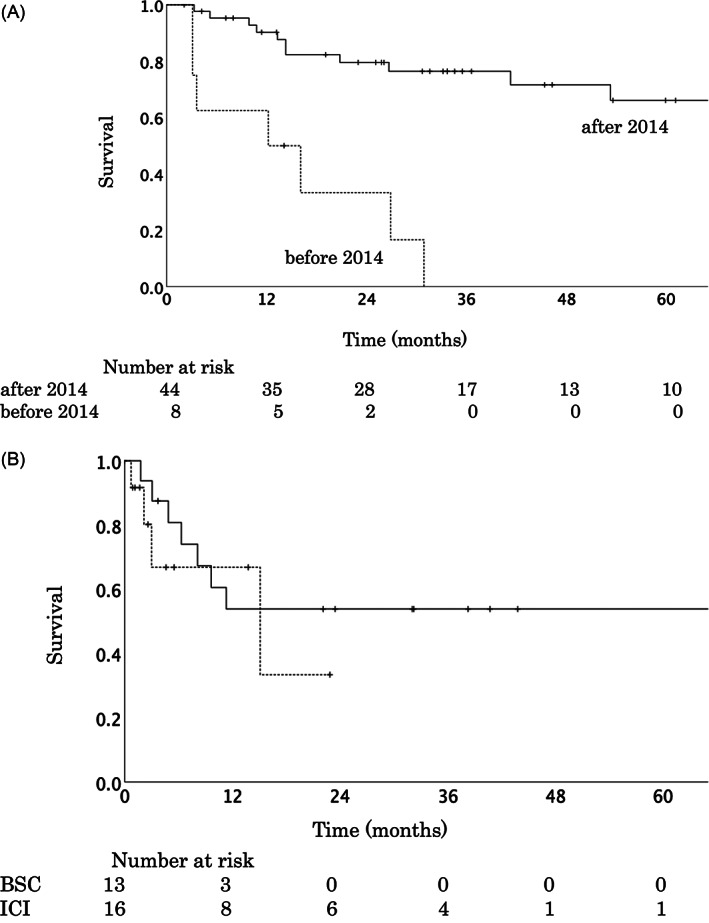
OS curve of patients with mucosal malignant melanoma treated with C‐ion RT with or without ICIs. (A) ICI agents were approved for treating melanoma in 2014 in Japan. The patients (*n* = 52) were divided into two groups based on whether they had been diagnosed before 2014 (*n* = 8) or after 2014 (n = 44). The 3‐year OS rates of those diagnosed before 2014 (dotted line) and those diagnosed after 2014 (straight line) were 0.0% and 76.4%, respectively (*p* = .000) (B) The 3‐year OS rates in the ICI group (*n* = 16) (straight line) and BSC group (*n* = 13) (dotted line) were 53.8% and 0.0%, respectively (*p* = 0.482). This is the OS after the diagnosis of recurrence (any site) and treatment with C‐ion RT. BSC, best supportive care; C‐ion RT, carbon‐ion radiotherapy; ICI, immune checkpoint inhibitor; OS, overall survival.

Of the 52 patients included, eight were diagnosed before 2014 and 44 were diagnosed after 2014 (Figure 1). The rate of the 3‐year OS of patients diagnosed before and after 2014 were 0.0% and 76.4%, respectively (95% CI before 2014, 6.696%–23.437%; after 2014, 61.364%–90.837%) (p = .000) (Figure 3A). Of the 29 patients diagnosed after 2014 who experienced local recurrence or distant metastasis, 16 patients received ICI agents, while 13 patients received the best supportive care (BSC) (Figure 1). Of the 16 patients who received ICIs for the first site of progression, two patients had local recurrence, 15 had distant metastasis (bone, *n* = 6; lymph node, *n* = 4; liver, *n* = 3; lung, *n* = 2; brain, *n* = 1; paranasal sinus, *n* = 1; skin, *n* = 1; and multiple sites, *n* = 2), and one had local recurrence and distant lymph node metastasis simultaneously (Table 3). The ICI agents used were anti‐PD‐1 (nivolumab and pembrolizumab) and anti‐CTLA4 (ipilimumab). Eight patients survived (Table 3).

**TABLE 3:** Patient characteristics treated with Immune checkpoint inhibitor therapy and therapeutic outcomes.

Patient characteristics						Carbon ion radiotherapy				Immuno‐checkpoint inhibitor				
No.	Age	Sex	Performance status	Tumor site	T stage	Total dose (Gy(RBE))	DAV cycle	Local recurrence	Distant metastasis	Agent/dose	Time from after C‐ion RT to ICI administration (month)	ICI toxicity	Follow‐up period after recurrence	After ICI survival
1	55	M	0	paranasal sinus	4a	64	3	−	brain	anti‐PD1(nivolumab) / 240 mg × 10	3	−	11.3	Dead
2	66	F	1	Nasal cavity	4a	64	3	+	Lymph node	Anti‐PD1(nivolumab)/240 mg × 4	85.5	Gastrointestinal toxicity (G1)	6.3	Dead
3	62	M	1	nasal cavity	4a	64	0	−	Paranasal sinus	Anti‐PD1(nivolumab)/240 mg × 6	33.8	Hepatobiliary toxicity (G1)	43.8	Alive
4	71	F	0	Nasal cavity	4a	64	3	−	Bone	Anti‐PD1(nivolumab)/240 mg × 1	10.2	Pneumonitis (G5)	3.1	Dead
5	80	M	1	Nasal cavity	4a	64	3	−	Lung	Anti‐PD1(nivolumab)/240 mg × 7	12.9	Mucositis (G3), Dermatitis (G3)	40.7	Alive
6	68	M	1	Nasal cavity	3	64	3	−	Liver	Anti‐PD1(nivolumab)/240 mg × 3	30	−	3.7	Alive
7	72	M	1	Nasal cavity	4a	64	3	−	Bone	Anti‐CTLA4(ipilimumab)/136 mg × 1	12.7	Dermatitis (G1), Hepatobiliary (G1), toxicity (G1)	8.1	Dead
8	63	M	1	Nasal cavity	4a	64	3	−	Liver	Anti‐PD1(nivolumab)/240 mg × 88	16.8	Dermatitis (G1)	87	Dead
9	62	M	1	Nasal cavity	4a	64	3	−	Lymph node	Anti‐PD1(nivolumab)/240 mg × 4 + anti‐CTLA3(ipilimumab) / 191 mg × 4, anti‐PD1(nivolumab) / 240 mg × 57	38.8	Dermatitis (G1)	32	Alive
10	61	M	0	Nasal cavity	4a	64	3	+	None	anti‐PD1(nivolumab) / 240 mg × 3 + anti‐CTLA3(ipilimumab)/179 mg × 3, anti‐PD1(nivolumab)/240 mg × 17	8	−	38.3	Alive
11	32	M	1	Paranasal sinus	4b	64	3	−	Bone	Anti‐PD1(nivolumab) / 190 mg × 4	5.1	Hepatobiliary toxicity (G1), Gastrointestinal toxicity (G1)	4.9	Dead
12	66	F	1	Nasal cavity	4a	64	3	−	Bone	Anti‐PD1(pembrolizumab) / 138 mg × 4	17	Gastrointestinal toxicity (g1), Fatigue (G1)	9.6	Dead
13	62	M	0	Oral cavity	4a	57.6	3	−	Lymph node	Anti‐PD1(nivolumab)/240 mg × 4, 200 mg × 10	2.4	Hypothyroidism (G2)	32.2	Alive
14	83	F	1	Nasal cavity	4a	64	0	−	Lung, liver, bone	Anti‐PD1(nivolumab) / 240 mg × 2	3.4	−	1.8	Dead
15	78	M	1	Nasal cavity	X	64	3	−	Lymph node	anti‐PD1(pembrolizumab) / 200 mg × 18, 400 mg × 3	10.2	−	23.5	Alive
16	68	M	0	Nasal cavity	4a	64	3	−	Bone, skin	anti‐PD1(nivolumab) / 240 mg × 3	3	−	22.1	Alive

Abbreviations: C‐ion, carbon‐ion; CTLA‐4, a cytotoxic T‐lymphocyte‐associated antigen 4; DAV, dacarbazine, nimustine, and vincristine; F, female; G1, Grade 1; G2, Grade 2; G3, Grade 3; G5, Grade 5; ICI, immune checkpoint inhibitor; M, male; PD‐1, programmed death 1; RBE, relative biological effectiveness; RT, radiotherapy.

The 3‐year OS rates of the ICI group (*n* = 16) and BSC group (*n* = 13) were 53.8% and 0.0%, respectively (95% CI, ICI group, 28.174%–71.543%; BSC group, 6.982%–19.802%) (*p* = .482) (Figure 3B). The OS (Figure 3B) was calculated after the diagnosis of recurrence (any site) and treatment with C‐ion RT. There was no significant difference in the 3‐year OS rates between the ICI and BSC groups; however, there was no mortality after 1 year among patients who received ICIs (Figure 3B).

The observed ICI‐related adverse events are shown in Table 3. None of the adverse events associated with C‐ion RT were related to or exacerbated by ICI administration. Most adverse events were grade 1 gastrointestinal and hepatobiliary toxicities. Grade 3 adverse events included mucositis (*n* = 1) and dermatitis (*n* = 1) and Grade 5 adverse events included pneumonitis (*n* = 1) (Table 3). The nivolumab‐only group consisted of 12 of 16 patients, eight (66.7%) of whom developed any grade of adverse event, while two (16.7%) developed adverse events of grade 3 or higher (Table 3).

## 4 DISCUSSION

In this study, the 3‐year OS rate was 64.3% for all patients. Patients diagnosed with recurrence after 2014 were categorized based on whether they received ICIs or BSC. However, there was no significant difference between the groups. A retrospective multi‐center study of C‐ion RT by J‐CROS showed similar outcomes, with a 2‐year OS rate of 69.4% and 5‐year OS rate of 44.6%.^6^ However, in the J‐CROS study, it was unclear whether ICIs were used in combination with C‐ion RT. Nevertheless, a good prognosis was showed in the ICI study, with a 5‐year OS rate of 52% with the use of nivolumab plus ipilimumab for advanced melanoma.^9^ In this study, the administration of nivolumab‐only was used in 11 patients. A previous clinical trial showed good prognosis for the use of nivolumab‐only, with a 5‐year OS rate of 44%.^9^ Therefore, the use of ICI agents for recurrence of HN‐MM after C‐ion RT may have contributed to survival.

The 3‐year LC in the current study was 86.3%. Similar results were obtained in a multi‐center study by J‐CROS, in which the 2‐year LC rates were 83.9% and 5‐year LC rates were 72.3%.^6^ Another prospective study reported a 3‐year LC rate of 92.3%.^7^ In our study, no cases of local recurrence due to modification of CTV or PTV were identified. Therefore, the C‐ion RT had a good local effect while protecting the OAR, similar to the previous reports.^6^, ^7^ Good LC may indicate good OS. Furthermore, some studies reported a favorable response after a long duration of ICI treatment.^15^, ^16^ In the present study, good local efficacy for OS was maintained with ICI therapy, and no deaths were reported after 1 year in patients who received ICIs. Therefore, a good OS can be attributed to both a good local response to C‐ion RT and a durable response to ICIs.

In this study, adverse events were documented for both C‐ion RT and ICI therapy. Previous studies have reported unique adverse events for ICI.^17^, ^18^ ICI therapy does not appear to exacerbate the adverse events of C‐ion RT and vice versa. No patient experienced the exacerbation of adverse events related to C‐ion RT following ICI therapy (Table 3). At the minimum, C‐ion RT or ICI agents could be safely administered. One study concluded that combining RT with ICI agents did not increase the risk of grade 3 or higher adverse events.^19^ Furthermore, a few studies reported no increase in the rate of adverse events when ICIs were administered before or after C‐ion RT.^20^, ^21^

A recent study on in‐vivo melanoma models found that C‐ion irradiation suppressed myeloid‐derived suppressor cells while increasing the lymphoid cells to a greater extent than X‐rays did with equivalent RBE.^22^ C‐ion irradiation releases high mobility group box 1 (HMGB1) from several cell lines of cancer, with the effect increasing with an increase in linear energy transfer.^23^ HMGB1 plays a leading role in danger signaling to cell death by immune response. On the other hand, high expression of PD‐L1 (programmed death‐ligand 1) has been reported to be associated with the efficacy of ICI therapy.^24^ In the current study, 13 of the 16 patients received nivolumab. PD‐L1 expression is induced in tumors after C‐ion RT,^25^, ^26^, ^27^ which may contribute to the good prognosis against anti‐PD‐1/PD‐L1 antibodies. Therefore, PD‐L1 evaluation of recurrent cases may also be helpful in prediction of prognosis of salvage treatment by ICIs. This is consistent with the results of Sato et al.,^28^ who showed that PD‐L1 expression is enhanced by deoxyribonucleic acid damage signals. Summarily, radiation has been reported to stimulate the immune response. Therefore, C‐ion RT may be a better immune stimulator than X‐rays. Although there was a time lag between the C‐ion RT and the administration of ICIs in this study, theoretically, a further improvement in efficacy may be expected using the combination of ICIs as early as possible.

This study had a few limitations. Firstly, several combinations and dosages of ICIs were used, the effects and adverse events of which should be further examined. Secondly, this study was retrospective and involved patients were enrolled from a single center. Future prospective studies with a larger sample size and including a greater number of institutions should be carried out. Moreover, it will be important to standardize ICI protocols in a prospective study to evaluate the effect of ICIs.

In conclusion, no significant differences were found in the survival rate between C‐ion RT followed by ICI therapy and C‐ion RT alone; however, patients who used ICIs for a longer period of time tended to survive longer. Therefore, ICI may be promising for lymph node metastases and distant metastases in patients with localized disease controlled by C‐ion RT. In addition, the other novelty of this study was that patients who received ICIs and C‐ion RT did not show a higher incidence of adverse events compared to patients treated with ICIs alone, and the possibility of combination therapy is clinically significant. As for the potential indications, the safety of the treatment has been confirmed in this report, and it has the potential to be fully translated into clinical practice.

## AUTHOR CONTRIBUTIONS

**Atsushi Musha:** Conceptualization (lead); data curation (lead); formal analysis (lead); funding acquisition (lead); methodology (lead); project administration (lead); writing – original draft (lead); writing – review and editing (equal). **Nobuteru Kubo:** Conceptualization (supporting); data curation (supporting); formal analysis (supporting); methodology (supporting); project administration (supporting); writing – original draft (supporting); writing – review and editing (supporting). **Hidemasa Kawamura:** Conceptualization (supporting); data curation (supporting); formal analysis (supporting); methodology (supporting); project administration (supporting); writing – original draft (supporting); writing – review and editing (supporting). **Naoko Okano:** Conceptualization (supporting); data curation (supporting); formal analysis (supporting); methodology (supporting); project administration (supporting); writing – original draft (supporting); writing – review and editing (supporting). **Hiro Sato:** Data curation (supporting); writing – original draft (supporting); writing – review and editing (supporting). **Kohei Okada:** Data curation (supporting); writing – original draft (supporting); writing – review and editing (supporting). **Kento Tomizawa:** Data curation (supporting); formal analysis (supporting); writing – original draft (supporting); writing – review and editing (supporting). **Norichika Ota:** Data curation (supporting); formal analysis (supporting); writing – original draft (supporting); writing – review and editing (supporting). **Akiko Adachi:** Data curation (supporting); formal analysis (supporting); writing – original draft (supporting); writing – review and editing (supporting). **Masato Shino:** Writing – review and editing (supporting). **Osamu Nikkuni:** Writing – review and editing (supporting). **Shota Ida:** Writing – review and editing (supporting). **Katsuyuki Shirai:** Methodology (supporting); project administration (supporting); writing – original draft (supporting); writing – review and editing (supporting). **Jun‐ichi Saitoh:** Methodology (supporting); project administration (supporting); writing – original draft (supporting); writing – review and editing (supporting). **Satoshi Yokoo:** Writing – review and editing (supporting). **Kazuaki Chikamatsu:** Writing – review and editing (supporting). **Tatsuya Ohno:** Data curation (supporting); formal analysis (supporting); writing – original draft (supporting); writing – review and editing (supporting).

## FUNDING INFORMATION

This work was supported by JSPS KAKENHI [grant Number 21K07693], the Takeda Science Foundation, and the Uehara Memorial Foundation. The funding sources had no involvement in the study design; in the collection, analysis and interpretation of data; in the writing of the report; and in the decision to submit the article for publication.

## CONFLICT OF INTEREST STATEMENT

The authors have stated explicitly that there are no conflicts of interest in connection with this article.

## ETHICS STATEMENT

This study was approved by the Gunma University Review Board (trial approval number: HS2021‐238) and carried out in accordance with the Declaration of Helsinki.

## PATIENT CONSENT STATEMENT

All study participants provided informed consent before treatment.

## Data Availability

The dataset generated and/or analyzed during the current study is not publicly available because it contains personal information, but anonymized data are available from the corresponding author on reasonable request.
